# Supplementary material for: Modeling glioblastoma heterogeneity as a dynamic network of cell states
Source: Mol Syst Biol. 2021 Sep 16;17(9):e10105. doi: 10.15252/msb.202010105 (PMC8444284; doi:10.15252/msb.202010105)
Supplement: Supplementary file 6 — Source Data for Figure 5 [file MSB-17-e10105-s004.zip › Figure5A_sourcedata/GSEA_3017/nosigdb_stateB.GseaPreranked.1622795021725/LARSSON_STATE_6.html]

Details for gene set LARSSON\_STATE\_6[GSEA]

|  || Dataset | state43017 |
| Phenotype | NoPhenotypeAvailable |
| Upregulated in class | na\_neg |
| GeneSet | LARSSON\_STATE\_6 |
| Enrichment Score (ES) | -0.4834829 |
| Normalized Enrichment Score (NES) | -2.63685 |
| Nominal p-value | 0.0 |
| FDR q-value | 0.0018215814 |
| FWER p-Value | 0.002 |
Table: GSEA Results Summary

  

Fig 1: Enrichment plot: LARSSON\_STATE\_6      
 Profile of the Running ES Score & Positions of GeneSet Members on the Rank Ordered List

  

| PROBE | GENE SYMBOL | GENE\_TITLE | RANK IN GENE LIST | RANK METRIC SCORE | RUNNING ES | CORE ENRICHMENT || 1 | GFAP |  |  | 132 | 0.487 | -0.1163 | No |
| 2 | CRYAB |  |  | 208 | 0.409 | -0.1655 | No |
| 3 | HEY1 |  |  | 253 | 0.376 | -0.1771 | No |
| 4 | TUBB2A |  |  | 296 | 0.351 | -0.1891 | No |
| 5 | ID3 |  |  | 389 | 0.311 | -0.2737 | No |
| 6 | ID1 |  |  | 390 | 0.311 | -0.2341 | No |
| 7 | FABP7 |  |  | 414 | 0.302 | -0.2267 | No |
| 8 | OLFM2 |  |  | 481 | 0.285 | -0.2796 | No |
| 9 | DKK3 |  |  | 549 | 0.273 | -0.3354 | No |
| 10 | PBX1 |  |  | 567 | 0.270 | -0.3240 | No |
| 11 | TUBA1A |  |  | 686 | -0.263 | -0.4500 | Yes |
| 12 | METRN |  |  | 695 | -0.273 | -0.4261 | Yes |
| 13 | SOX4 |  |  | 712 | -0.305 | -0.4088 | Yes |
| 14 | NEAT1 |  |  | 714 | -0.308 | -0.3709 | Yes |
| 15 | CTXN1 |  |  | 715 | -0.309 | -0.3316 | Yes |
| 16 | MARCKSL1 |  |  | 716 | -0.311 | -0.2919 | Yes |
| 17 | PTMS |  |  | 720 | -0.333 | -0.2536 | Yes |
| 18 | PNRC1 |  |  | 726 | -0.348 | -0.2161 | Yes |
| 19 | MXD4 |  |  | 731 | -0.384 | -0.1725 | Yes |
| 20 | FABP5 |  |  | 759 | -1.641 | -0.0000 | Yes |
Table: GSEA details [plain text format]

  

Fig 2: LARSSON\_STATE\_6: Random ES distribution      
 Gene set null distribution of ES for **LARSSON\_STATE\_6**
